# Persistent hyperglycemia modulates gut immune function and microbiota in rats

**DOI:** 10.1186/s40560-015-0101-8

**Published:** 2015-07-23

**Authors:** Katsuya Mori, Takeshi Suzuki, Toru Igarashi, Kei Inoue, Takashi Asahara, Koji Nomoto, Hiroyuki Seki, Takashige Yamada, Shizuka Minamishima, Shizuko Kosugi, Nobuyuki Katori, Hiroshi Morisaki

**Affiliations:** Department of Anesthesiology, Keio University School of Medicine, 35 Shinanomachi, Shinjuku-ku, Tokyo 160-8582 Japan; Yakult Central Institute for Microbiological Research, 5-11 Izumi, Kunitachi-shi, Tokyo 186-0012 Japan

**Keywords:** Gut barrier function, Pro-inflammatory cytokine, CD4^+^ T lymphocyte subsets, Mesenteric lymph nodes

## Abstract

**Background:**

Since hyperglycemia-induced cellular dysfunction could be associated with alterations of the immune system, we tested the hypothesis that hyperglycemia augments the aberrant immune responses such as inflammation and differentiation of CD4^+^ T lymphocytes in the mesenteric lymph nodes (MLNs), and induces alterations of microbiota both under physiological and pathological conditions.

**Methods:**

Male Wistar rats were randomly allocated into 4 groups: Control and Endotoxemia (lipopolysaccharide, LPS 1 mg/kg) with or without hyperglycemia. The hyperglycemia groups were administered glucose solution (10-40 %), while the normoglycemia groups were administered saline. Alterations of the mRNA expressions of inflammatory cytokines and CD4^+^ T lymphocyte transcriptional factor expressions in the MLNs, and those of the intestinal microbiota were analyzed at 24 hr.

**Results:**

Hyperglycemia was kept approximately 250–350 mg/dL during the 24 hr study period. At the end of 24 hr, hyperglycemia augmented the mRNA expressions of interleukin (IL)-1β and IL-6 in the MLNs, while both the helper T (Th) 2 and regulatory-T (Treg) transcriptional factors were simultaneously up-regulated under non-endotoxemic condition. LPS injection significantly modulated the obligate anaerobe bacterial populations of the *Bacteroidetes* class, and altered the population sizes of the *Clostridium perfringens* and the *Bacteroides fragilis* subgroup. Hyperglycemia did not enhance these alterations of the microbiota evoked by LPS, although it did modify the bacterial populations of the *L. reuteri* subgroup and staphylococci in healthy condition without endotoxemia.

**Conclusions:**

The present study indicates that both gut immune function and microbiota are significantly modulated by persistent hyperglycemia.

**Electronic supplementary material:**

The online version of this article (doi:10.1186/s40560-015-0101-8) contains supplementary material, which is available to authorized users.

## Background

Critically ill patients with persistent hyperglycemia are well known to show higher mortality rates, possibly due to the deterioration of a variety of cell functions [1, 2]. Although the exact mechanisms remain to be fully clarified, functional alterations of the immune system and related cells could, at least in part, be responsible for the organ dysfunctions and poor outcomes of patients with hyperglycemia [2, 3]. For example, hyperglycemia apparently alters the functions of immune cells such as monocytes, neutrophils and T or B lymphocytes [2, 4, 5]. In addition, hyperglycemia, by itself, is known to augment the production of inflammatory cytokines such as tumor necrosis factor-α (TNF-α) and interlukin-6 (IL-6) [6].

Maintenance of the intestinal homeostasis and function is very important issue to improve the prognosis of critically ill patients, since intestinal barrier dysfunction has been shown to contribute to the deterioration of the host immune function. Among the gut immune systems, gut-associated lymphoid tissue (GALT) is of great consequence, not only in relation to regulation of the intestinal homeostasis, but also to the maintenance of systemic immunity [7]. In particular, mesenteric lymph nodes (MLNs) of GALT are considered as the key tissues for antigen-driven priming, activation and polarization of naive T cells towards several T cell subpopulations, such as helper T cell 1 (Th1), 2 (Th2), 17 (Th17) and regulatory T cells (Treg) [7, 8]. As apparent overproduction of inflammatory cytokines has been found in the MLNs of critically ill conditions [9], it is likely that the normal MNL function could be complicated in critically ill patients. On the other hand, an enormous amount of bacteria, estimated at approximately 10^12^ commensal bacteria covering over 1,000 species, inhabit and together constitute independent bacterial ecosystems, known as microbiota, in the gut [10]. Although the microbiota contribute to the development of the host immune potential [7], its alterations in quality and quantity, called dysbiosis, have been shown to play substantial roles in gut inflammation and metabolic disease [11], and are associated with organ dysfunctions and poor outcomes in critically ill patients through causing gut barrier dysfunction [12].

We previously demonstrated that excessive hyperglycemia at levels of over 400 mg/dL for short-term periods, like 3 hr, did not evoke alterations of the gut microbiota, but apparently induced gut barrier dysfunction through TNF-α-dependent pathways [13]. However, it remains to be determined if longer time period of hyperglycemia induce the change of the immune status of MLNs and the alterations of the microbiota, especially in the critically ill condition.

In the present study, we therefore hypothesized that persistent hyperglycemia for 24 hrs up-regulated the pro-inflammatory cytokines, and thereby promoted the differentiation of CD4^+^ lymphocytes to Th1 and Th17, which posses the ability of inducing inflammatory responses, in MLNs. Besides, we examined whether these alterations of the immune status in MLNs were followed by the disruption of the microbiota homeostasis; that is, increase of pathogens and decrease of probiotics. Furthermore, we proposed the 2x2 study design; control and hyperglycemia group with/without eodotoxemia, to evaluate the effect of disease condition on the change induced by hyperglycemia. We expected that endotoxemia augmented the effect of hyperglycemia.

## Methods

This study protocol was approved by the Animal Care and Committee of Keio University School of Medicine (KI002001) in accordance with the National Institute of Health guideline.

### Animal preparation

Male Wistar rats (6–7 weeks), weighting 350–400 g, were fed standard chow and studied after an acclimation period of 3 to 7 days at our laboratory. Under isoflurane anesthesia in oxygen, the jugular vein and carotid artery were cannulated (PE50; Intermedic, Sparks, MD). Jugular vein was used for continuous infusion of glucose solution, whereas arterial line was used for continuous monitoring and blood sampling described below. After the preparatory surgery, the animals were placed in a metabolic cage that allowed for awakening and stabilization for 1 hr.

### Study protocol

After the preparatory surgery and 1-hr stabilization period, 20 rats were randomly assigned to either the Control group (C: *n* = 10) or the Endotoxemia (E: *n* = 10) group, the animals in the latter of which were administered LPS (1 mg/kg) by intravenous injection. Based on our pilot study data, we chose this dose of LPS in which the mortality rate at 48 hrs was approximately 10-15 % without hyperglycemia and 20-30 % with hyperglycemia. In other words, we intended to make our endotoxemia model not lethal but moderately damaged to mimic disease condition. Both groups were further divided into 2 subgroups (*n* = 5, each group): the Normoglycemia group (N: 80–150 mg/dL), administered normal saline, and the Hyperglycemia group (H: 250–350 mg/dL), administered glucose solution, resulting in four different groups (CN, CH, EN, and EH group). All solutions were infused at the rate of 10 ml/kg/hr throughout the 24 hr study period. In our pilot study, we confirmed that such infusion rate did not alter arterial pH and electrolytes significantly at least for 24 hrs. Blood sample (10 μL) was obtained at 0, 3, 6, 12, and 24 hr study period for measurement of blood glucose. In the Hyperglycemia group, all rats received 20 % of glucose infusion at base line, and then, the concentration was adjusted according to the blood glucose levels measured at 3, 6, and 12 hr time-points during the study period. If blood glucose level was more than 350 or 500 mg/dL, the infusion concentration was reduced by 5 % or 10 % respectively. On the other hand, if blood glucose was less than 200 or 150 mg/dL, the infusion concentration was increased by 5 % or 10 % respectively (maximum 40 %, minimum 10 % of glucose infusion). Since one animal in the EH group died before the end of the study period, we added one rat in the EH group so that the number of animals in each group was equal (*n* = 5). At 24 hr, the MLNs were excised thorough laparotomy and the adipose tissue around it was removed for the measurement of inflammatory cytokine (tumor necrosis factor-α [TNF-α], interleukin-1β [IL-1β] and IL-6) mRNAs and levels of transcriptional factors of CD4^+^ T lymphocyte subsets (Th1, Th2, Th17 and Treg). Then, all tissues were incubated in RNAlater solution (Ambion Inc., Austin, TX, USA) overnight at 4 °C and stored at −80 °C until the mRNA measurement by semi-quantitative real time-polymerase chain reaction (Semi-quantitative RT-PCR).

To clarify the alterations of the gut microbiota, another series of experiments were performed: Following the same methods described above, 26 rats were randomly divided into the same 4 groups (CN: *n* = 6, CH: *n* = 7, EN: *n* = 6, EH: *n* = 7) and received the same treatment during the next 24 hr study period. One rat was added to the EH group, since one rat in this group died before the end of the study period. After laparotomy under anesthesia, the contents of colon were harvested from the whole colon, and sampled and weighed before suspension in RNAlater solution (Ambion Inc., Austin, TX, USA) and then stored at 4 °C until the analyses described below.

### Preparation of total RNA for evaluation of the cytokines and CD4^+^ T lymphocyte subsets

We performed semi-quantitative RT-PCR to examine the mRNA level of each of the inflammatory cytokines and CD4^+^ T lymphocyte master genes in the MLNs. The total RNA from the MLNs was extracted using RiboPure^TM^ kit (Ambion, CA, USA). Briefly, 50 mg of minced MLNs was transferred to 1.5-ml sterile tubes containing microbeads. Then, 1-ml TRI regent (Ambion, CA, USA), a monophasic solution containing phenol and guanidine thiocyanate, was added to the tubes, followed by homogenization using a tissue homogenizer (Micro Smash-100R, Tomy, Japan) at 2,000 x g for 2 min at 4 °C. After the homogenization, each sample was centrifuged at 4,500 x g for 10 min at 4 °C and the supernatants were transferred to 1.5-ml sterile tubes. Then, 100 μL of 1-Bromo-3-Chloropropane, a phase separation reagent, was added to the supernatants, followed by incubation and centrifugation at 4,500 x g for 10 min at 4 °C, and transfer of the aqueous phase to 1.5-mL sterile tubes, to which 200 μL of 100 % ethanol was added. Thereafter, the samples were transferred into 1.5-mL sterile tubes with a filter cartridge, and centrifuged at 8,000 rpm for 10 min at 4 °C. The filter cartridges containing total RNA were washed twice with wash solution, followed by addition of 100 μL of elution buffer and centrifugation for 30 sec. The quantity and quality of the eluted total RNA were measured using the NanoDrop 1000A spectrophotometer (Thermo Fisher Scientific-NanoDrop products, DE, USA), followed by dilution of the RNA samples with nuclease-free water (Ambion, CA, USA). To remove contaminating DNA from the isolated RNA, DNase treatment was performed using a DNA-free kit (Ambion, CA, USA). The complementary DNA (cDNA) synthesis from the total RNA was performed using the ReverTra Ace qPCR RT kit (Toyobo, Tokyo, Japan) with random primers, in accordance with the instructions supplied by the manufacturer.

### Determination of the mRNA expression levels of cytokines and CD4^+^ T lymphocyte master genes by semi-quantitative-RT-PCR

The target gene expressions were analyzed by semi-quantitative RT-PCR using TaqMan-based Applied Biosystem gene expression assays (Applied Biosystem, CA, USA). The primers and probes designed for this system were as follows; Rn9999017_m1 (*Tnf*) for TNF-α, Rn00580432_m1 (*Il1b*) for IL-1β, Rn01410330_m1 (*Il6*) for IL-6, Rn01461633_m1 (*Tbx21*) for Th1 transcriptional factors, Rn00484683_m1 (*Gata3*) for Th2 transcriptional factors, Rn01533717_g1 (*Rorc*) for Th17 transcriptional factors, Rn01525092_m1 (*Foxp3*) for Treg transcriptional factors, and Rn00607869_m1 (*Actb*) for β-actin. Quantification was normalized to the endogenous expression of the β-actin gene. The reaction mixture and PCR cycles were adjusted according to the manufacturer’s instructions (Applied Biosystem, CA, USA). The data were analyzed using the OneStep Software (Applied Biosystem, CA, USA). After determining the comparative threshold cycle (*C*_*T*_) values for β-actin as reference, the relative quantifications were carried out using the 2^-ΔΔ*Ct*^ method and standardized by the *C*_*T*_ value for β-actin. The data were expressed as mean ± standard deviation.

### Isolation of total RNA for the microbiota analysis and bacterial count by quantitative RT-PCR

After the intestinal contents of the colon were homogenized using a mortar on ice, an aliquot of fecal homogenate (20 μL) was added to 1 mL sterilized PBS solution and centrifuged at 5,000 x g for 10 min. The supernatant was discarded and RNA was isolated using a modification of the guanidinium thiocyanate-phenol-chloroform extraction method [14, 15]. To quantify the bacteria present in the samples, we examined the gut microbiota composition by the 16S rRNA-targeted quantitative RT-PCR kit, Yakult Intestinal Flora-SCAN (YIF-SCAN®) [14, 15]. Quantitative RT-PCR was conducted in a one-step reaction using a Qiagen OneStep RT-PCR kit (Qiagen GmbH, Hilden, Germany) and SYBR green method (Molecular Probes, Eugene, OR). The products of reaction mixture, reverse transcription and PCR cycles were assessed as previously described [14, 15]. A standard curve was generated using the *C*_*T*_ values of quantitative RT-PCR data and the corresponding cell counts, determined microscopically with 4,6-diamidino-2-phe-nylindole (DAPI) (Vector Laboratories, Burlingame, CA) staining methods, of the dilution series of the standard strains. In the present study, we measured the various types of bacteria in the gut microbiota by using specific primer sets, including major types of bacteria in the intestine, such as Enterococcus, and Enterobacteriaceae to which E.coli belong. To determine the number of bacteria present in the samples, 3 serial 10-fold dilutions of the extracted RNA sample (corresponding to 1/2,000, 1/20,000, and 1/200,000 of the amount of RNA extracted from 20 mg of colon contents) were used for the quantitative RT-PCR, and the *C*_*T*_ values in the linear range of the assay were applied to the standard curve generated in the same experiment to obtain the corresponding bacterial cell counts in each nucleic acid sample. The specificity of the quantitative*-*RT-PCR assay using group- or species-specific primers was determined as described previously [14, 15]

### Statistical analyses

All data were expressed as mean ± SD after confirmation of normal distribution. The repeated-measures data of blood glucose obtained at various time-points were analyzed by mixed-effects analysis of variance (ANOVA) using the statistical software package, EXCEL-Longitudinal Data Analysis, Ver1.0 for Windows (ESUMI, Tokyo, Japan). Then, *Post-hoc* test was performed using Tukey’s test. Other data, such as the mRNA levels detected by RT-PCR were analyzed by two-way ANOVA for interaction between two main effects of endotoxemia (Factor A; C vs E) and hyperglycemia (Factor B; N vs H). Ninety five percent of confidence interval for each four factors (C, E, N, and H) was also presented. When the interaction between two factors didn’t exist, independent effects of these two factors were presented. If the interaction were detected, comparison of two groups were performed using Student’s t-test separately in the Control group (CN vs CH) and the Endotoxemia group (EN vs EH) to elucidate the effect of hyperglycemia in both healthy and endotoxemic condition. The statistical analyses for mRNA were performed using SPSS/17.0 J (SPSS Inc, Chicago, IL). If mRNA was not detectable by RT-PCR, the value was regarded as zero. Differences at two-sided p-values of less than 0.05 were considered to be statistically significant.

## Results

### Changes of the blood glucose concentrations during the study period

**Fig. 1:**
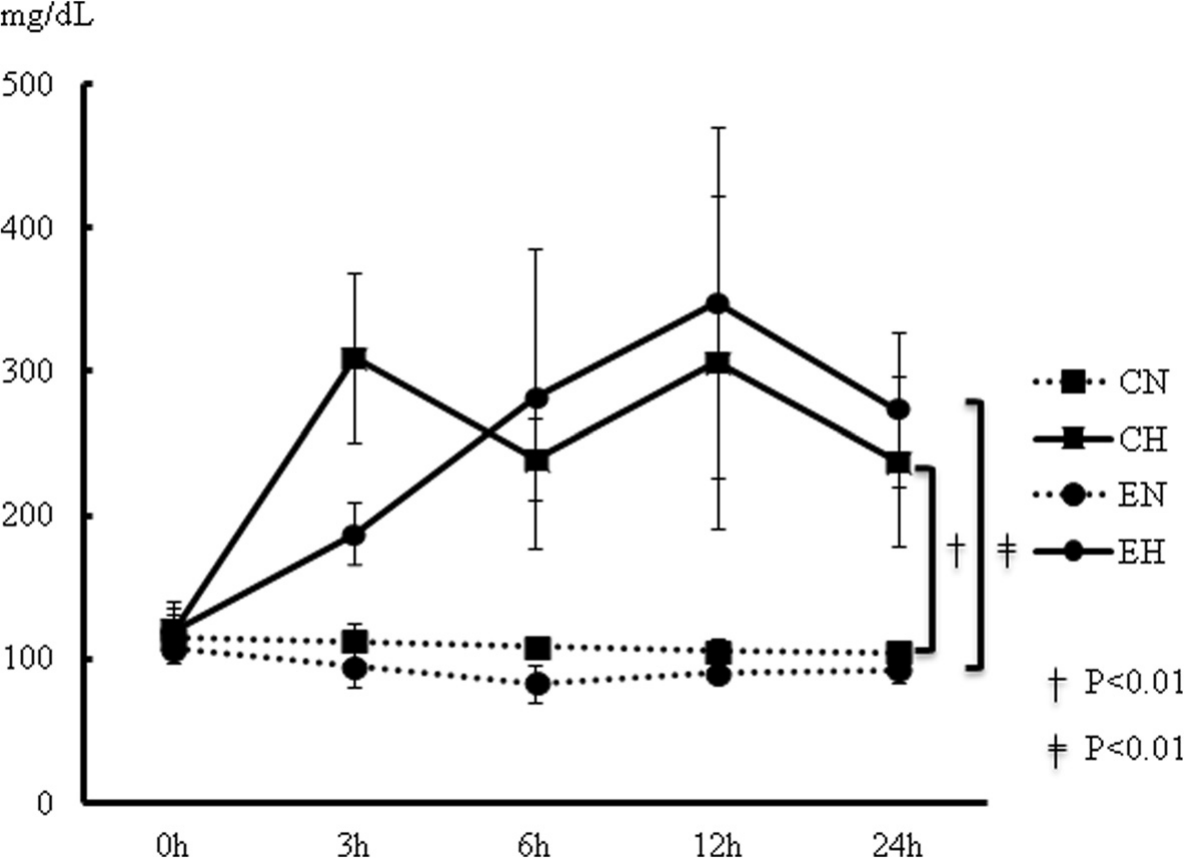
Changes of blood glucose concentrations during 24 hr study period. Comparison between four groups was performed by mixed-effects analysis of variance (ANOVA) followed by Tukey’s *post hoc* test. Abbreviation; CN: control with normoglycemia group, CH: control with hyperglycemia group, EN: endotoxemia with normoglycemia group, EH: endotoxemia with hyperglycemia group. † *P* < 0.01: CH *versus* CN, ‡: *P* < 0.01 EH *versus* EN. Data are expressed as mean ± SD

Figure 1 indicates the changes of the blood glucose levels over the 24 hr study period. While the blood glucose levels in both the normoglycemic (CN and EN) groups were approximately in the range of 80 mg/dL to 120 mg/dL, the hyperglycemic groups (CH and EH) showed persistent hyperglycemia of approximately 250 to 350 mg/dL throughout the 24 hr study period.

### Alterations of the mRNA expressions of the inflammatory cytokines and CD4^+^ T lymphocyte subsets in the MLNs

**Fig. 2:**
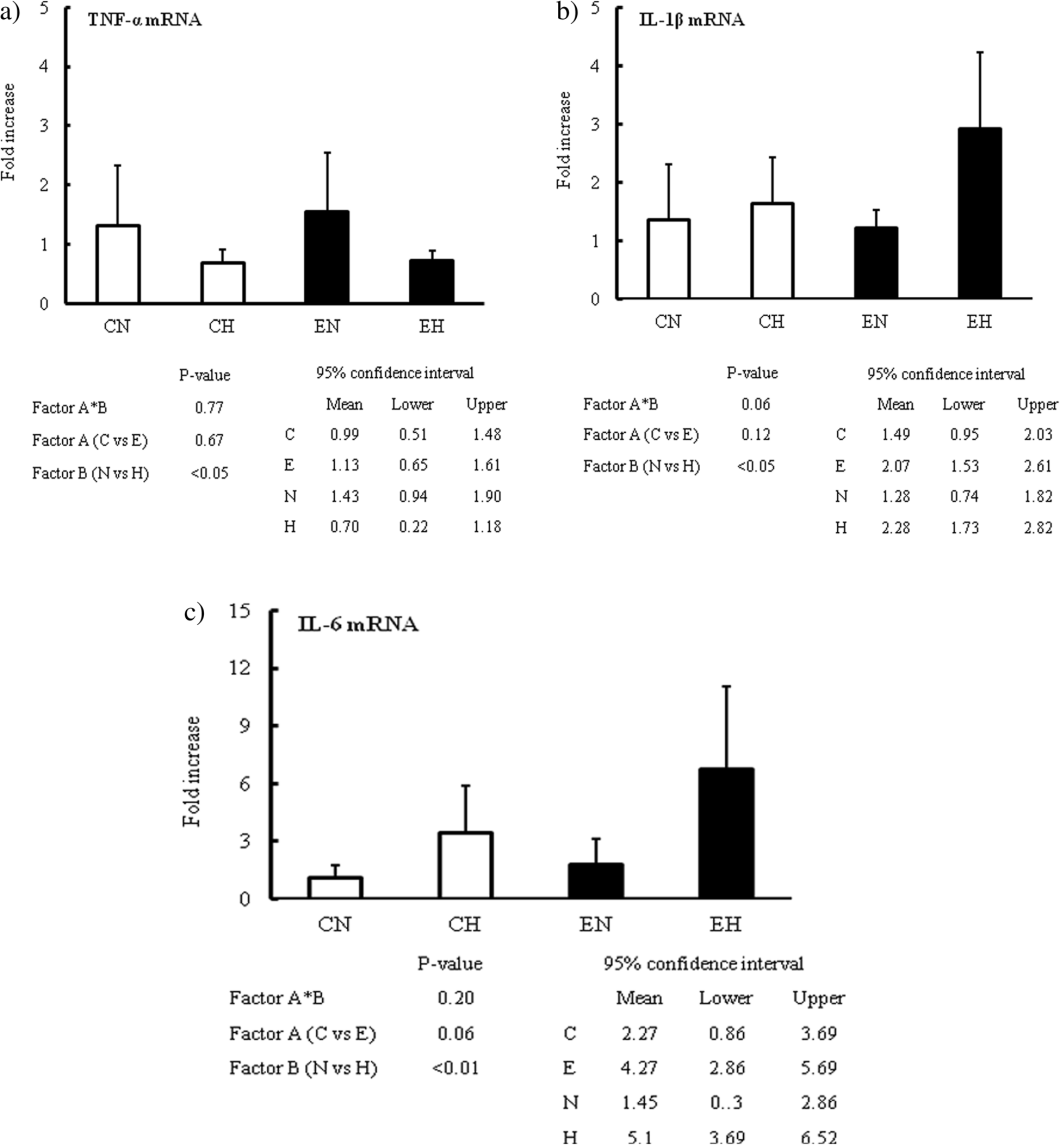
Changes of TNF − α, IL-1β and IL-6 mRNA expression in MLNs. a) Expression of TNF-α mRNA at 24 hr study periods in MLNs. b) Expression of IL-1β mRNA at 24 hr study periods in MLNs. c) Expression of IL-6 mRNA at 24 hr study periods in MLNs. Inflammatory cytokines were analyzed by two-way ANOVA for interaction between factor A (control *versus* endotoxemia) and factor B (normoglycemia *versus* hyperglycemia). Ninety five percent confidence intervals for each four factors were also presented. If the interaction between two factors didn’t exist, independent effects of these two factors were confirmed. If the interaction existed, comparison of two groups were performed using Student’s t-test separately in the Control group (CN vs CH) and the Endotoxemia group (EN vs EH). Abbreviation; CN: control with normoglycemia group, CH: control with hyperglycemia group, EN: endotoxemia with normoglycemia group, EH: endotoxemia with hyperglycemia group. Data are expressed as mean ± SD. White and black bars indicate control and endotoxemia group, respectively

No interaction between hyperglycemia and endotoxemia was found in the context of the expression of TNF-α, IL-1β or IL-6 mRNA in the MLNs. Furthermore, significant differences were observed between the hyperglycemic and normoglycemic groups in the expression levels of all of these cytokines. For instance, hyperglycemia *per se*, regardless of the presence/absence of concurrent endotoxemic insult, depressed the expression of TNF-α mRNA, but augmented the IL-1β and IL-6 mRNA expression in MLNs (Fig. 2a, b and c).

**Fig. 3:**
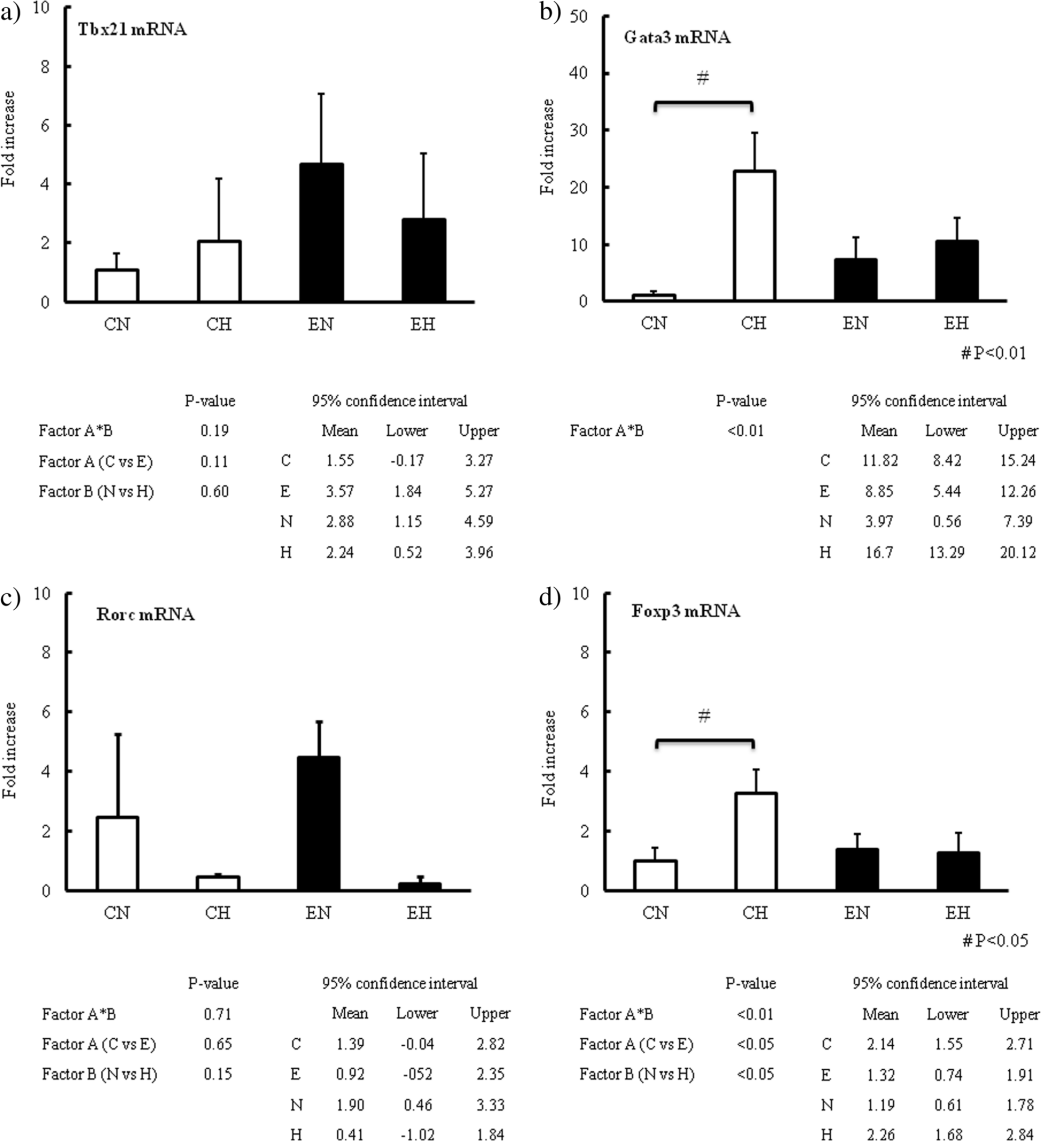
Changes of Tbx21, Gata3, Rorc and Foxp3 mRNA expression in MLNs. a) Expression of Tbx21 mRNA at 24 hr study period in MLNs. b) Expression of Gata3 mRNA at 24 hr study period in MLNs. c) Expression of Rorc mRNA at 24 hr study period in MLNs. d) Expression of Foxp3 mRNA at 24 hr study period in MLNs. Four types of transcriptional factors for CD4+ T lymphocytes were analyzed by two-way ANOVA for interaction between factor A (control *versus* endotoxemia) and factor B (normoglycemia *versus* hyperglycemia). Ninety five percent confidence intervals for each four factors were also presented. If the interaction between two factors didn’t exist, independent effects of these two factors were confirmed. If the interaction existed, comparison of two groups were performed using Student’s t-test separately in the Control group (CN vs CH) and the Endotoxemia group (EN vs EH). Abbreviation; CN: control with normoglycemia group, CH: control with hyperglycemia group, EN: endotoxemia with normoglycemia group, EH: endotoxemia with hyperglycemia group. Data are expressed as mean ± SD. White and black bars indicate control and endotoxemia group, respectively. P values described in the figure indicates those obtained by *post hoc* test

In the analysis of the transcriptional factors of CD4^+^ T lymphocytes, the interaction between endotoxemia and hyperglycemia was not statistically significant in the effect on Tbx21 (Fig. 3a) or Rorc (Fig. 3c), but was in the effect on Gata3 (Fig. 3b) and Foxp3 (Fig. 3d). There were no independent significant effects of endotoxemia or hyperglycemia on Tbx21 and Rorc mRNA. Regarding Gata3 and Foxp3 mRNA, hyperglycemia induced a significant augmentation of Gata3 (Fig. 3c, *p* < 0.01) and Fox3 mRNA (Fig. 3d, *p* < 0.05) in the Control group only, suggesting that hyperglycemia could induce the transcription of Gata3 and Foxp3 as representatives of Th2 and Treg in the MLNs in the healthy condition, not the endotoxemic condition.

### Alterations of the intestinal microbiota

To clarify whether persistent hyperglycemia evoked by exogenous glucose infusion for 24 hrs modulates the intestinal microbiota differently in the presence or absence of endotoxemia, we evaluated the changes in the populations of the obiligate/facultative anaerobes and aerobes by quantitative RT-PCR. The classification of bacteria we evaluated in this study is shown in the Additional file 1: Figure S1.

**Fig. 4:**
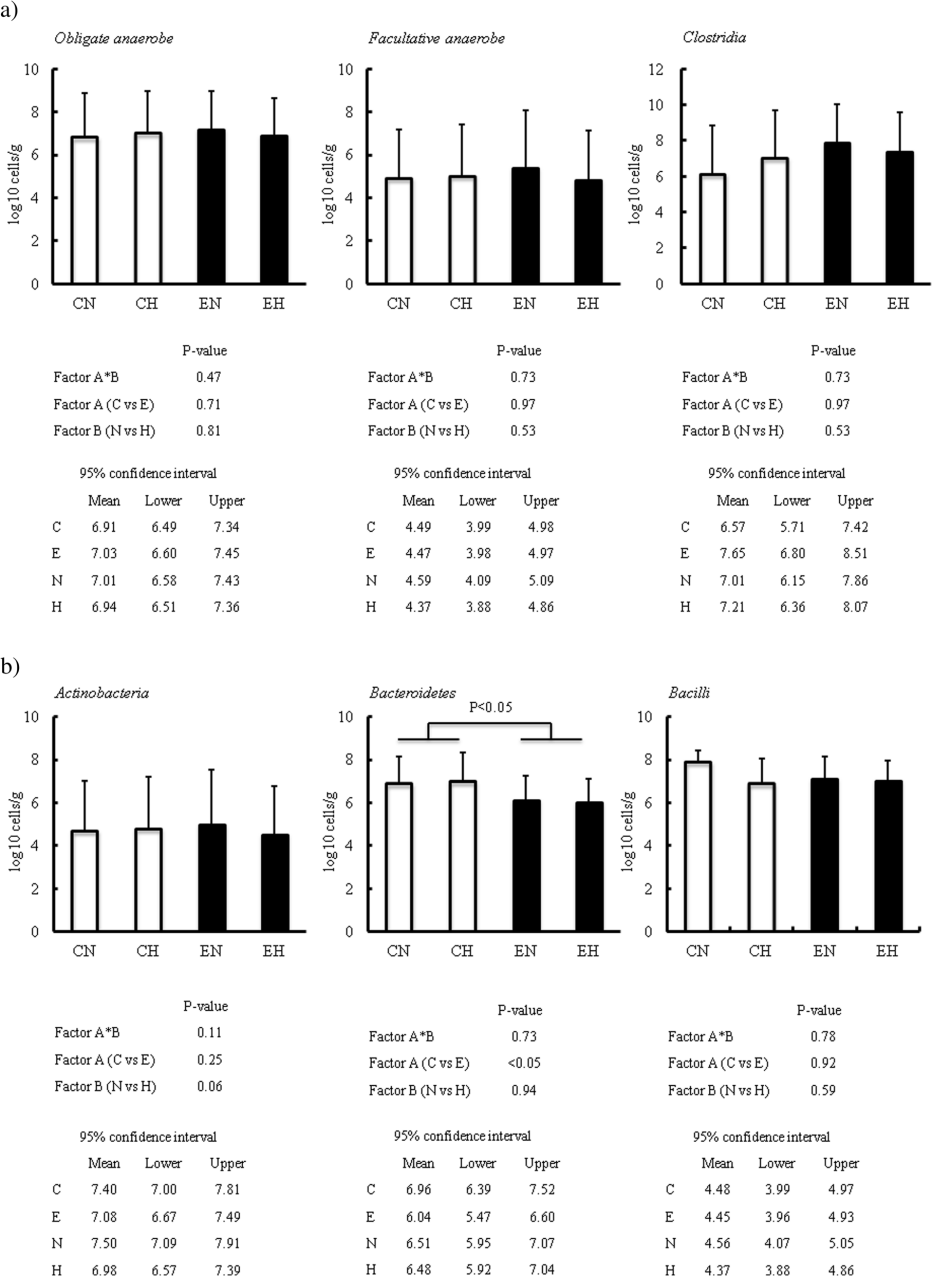
Alterations of obligate and facultative anaerobes, individual bacteria classes in colon contents. Alterations of obligate and facultative anaerobes, individual bacteria classes in colon contents were analyzed by two-way ANOVA for interaction between factor A (control *versus* endotoxemia) and factor B (normoglycemia *versus* hyperglycemia). Ninety five percent confidence intervals for each four factors were also presented. If the interaction between two factors didn’t exist, independent effects of these two factors were confirmed. If the interaction existed, comparison of two groups were performed using Student’s t-test separately in the Control group (CN vs CH) and the Endotoxemia group (EN vs EH). The main effect of endotoxemic insult was found only in class *Bcteroidetes*. Abbreviation; CN: control with normoglycemia group, CH: control with hyperglycemia group, EN: endotoxemia with normoglycemia group, EH: endotoxemia with hyperglycemia group. Data are expressed as mean ± SD (log10 cells/g of colon contents). White and black bars indicate control and endotoxemia group, respectively

There were no significant differences in the total populations of obligate/facultative anaerobes and aerobes in the colon contents among the groups (Fig. 4). In the class level analysis, however, the independent main effect of endotoxemia was found on the *Bacteroidetes* class (decrease), even though hyperglycemia had no independent effects (Fig. 4). Table 1 shows the alteration of each type of bacteria in the colon microbiota, presented as the logarism_10_ cells/g of colon contents. In the lower-level analyses than the class level, endotoxemia induced the increase of *Clostridium perfringens* and the decrease of *Bacteroides fragilis* subgroups independently regardless of the existence of hyperglycemia, while hyperglycemia itself decreased the *Lactobacillus ruminis* subgroup without the interaction effect of endotoxemia. The interaction between the two factors (hyperglycemia and endotoxemia) was found on the *Lactobacillus reuteri* subgroup, *staphylococci*, *enterococci* and *Enterobacteriaceae* (Table 1). In the Control group (healthy condition), hyperglycemia induced the decrease of *Lactobacillus reuteri* subgroup, and the increase of *staphylococci*. On the other hand, hyperglycemia decreased both *enterococci* and *Enterobacteriaceae* in the Endotoxemia group (endotoxemic condition).

**Table 1:** Alteration of lower than class level bacteria in colon microbiota

	CN group (detection rate)	CH group (detection rate)	EN group (detection rate)	EH group (detection rate)	Factor A (C vs E)	Factor B (N vs H)	Factor A*B
Total bacterial counts	9.4 ± 0.2 (6/6)	9.8 ± 0.8 (7/7)	10.1 ± 0.4 (6/6)	9.7 ± 1.1 (7/8)	*P* = 0.43	*P* = 0.98	*P* = 0.31
Obligate anaerobe							
*Clostridium coccoides* group	7.8 ± 0.3 (6/6)	8.6 ± 0.5 (7/7)	9.1 ± 0.4 (6/6)	8.7 ± 1.1 (7/7)	*P* = 0.10	*P* = 0.67	*P* = 0.11
*C. leptum* subgroup	8.2 ± 0.5 (6/6)	8.9 ± 0.7 (7/7)	9.5 ± 0.3 (6/6)	8.8 ± 1.1 (7/7)	*P* = 0.17	*P* = 0.99	*P* = 0.12
*C. perfringens*	<2.3 (6/6)	3.5 ± 1.6 (4/7)	5.1 ± 1.2 (6/6)	4.7 ± 1.1 (6/7)	*P* < 0.01	*P* = 0.39	*P* = 0.12
*Bifidobacterium*	7.7 ± 0.6 (6/6)	6.4 ± 1.2 (7/7)	6.4 ± 1 (5/6)	6.5 ± 0.9 (6/7)	*P* = 0.09	*P* = 0.13	*P* = 0.06
*Atopobium cluste*r	8.1 ± 0.4 (6/6)	7.4 ± 1 (6/7)	7.7 ± 0.5 (6/6)	7.6 ± 0.8 (7/7)	*P* = 0.1	*P* = 0.16	*P* = 0.57
*Prevotella*	5.9 ± 1.1 (3/6)	6.4 ± 1.4 (4/7)	7.2 ± 0.4 (6/6)	6.6 ± 1.1 (7/7)	*P* = 0.12	*P* = 0.90	*P* = 0.24
*Bacteroides fragilis* group	7.9 ± 0.5 (6/6)	7.6 ± 1.2 (7/7)	<5.0 (0/6)	7.2 (1/7)	*P* < 0.01	*P* = 0.97	*P* = 0.44
Facultative anaerobe							
*L. gasseri* subgroup	9.1 ± 0.5 (6/6)	9.5 ± 1.2 (7/7)	9.9 ± 0.6 (6/6)	9.4 ± 1.3 (7/7)	*P* = 0.46	*P* = 0.97	*P* = 0.41
*L. brevis*	<2.3 (6/6)	2.9 ± 1 (2/7)	3.4 ± 0.8 (6/6)	3.0 ± 0.9 (3/7)	*P* = 0.08	*P* = 0.75	*P* = 0.19
*L. casei* subgroup	<3.2 (0/6)	<3.2 (0/7)	<3.2 (0/6)	<3.2 (0/7)	-	-	-
*L. fermentum*	<4.0 (0/6)	<4.0 (0/7)	<4.0 (0/6)	<4.0 (0/7)	-	-	-
*L. plantarum* subgroup	<2.9 (0/6)	<2.9 (0/7)	<2.9 (0/6)	<2.9 (0/7)	-	-	-
*L. reuteri* subgroup	7.7 ± 0.7 (6/6)	4.9 ± 2.8 (4/7) *	2.8 ± 0.9 (4/6)	2.8 ± 0.8 (5/7)	-	-	*P* < 0.05
*L. ruminis* subgroup	4.5 ± 0.8 (6/6)	4.4 ± 1.1 (6/7)	7.2 ± 1.3 (6/6)	5.3 ± 1.7 (7/7)	*P* = 0.07	*P* < 0.01	*P* = 0.05
*L. sakei* subgroup	2.5 ± 0.4 (3/6)	2.8 ± 0.6 (6/7)	2.9 ± 0.7 (5/6)	2.9 ± 0.8 (5/7)	*P* = 0.38	*P* = 0.69	*P* = 0.57
*Staphylococcus*	5.1 ± 0.7 (6/6)	6.7 ± 1 (7/7) **	6.3 ± 0.7 (6/6)	5.4 ± 1.4 (6/7)	-	-	*P* < 0.01
*Enterococcus*	6.7 ± 1 (6/6)	7 ± 0.7 (7/7)	7.8 ± 0.7 (6/6)	6.8 ± 0.8 (7/7) ***	-	-	*P* < 0.05
*Enterobacteriaceae*	6.6 ± 1 (6/6)	7 ± 1.5 (6/7)	8.8 ± 0.3 (6/6)	7.6 ± 0.6 (7/7) ****	-	-	*P* < 0.05
Aerobes							
*Pseudomonas* species	<3.0 (0/6)	<3.0 (0/7)	<3.0 (0/6)	<3.0 (0/7)	-	-	-

The numbers are presented as logalithm_10_ cells/g of colon contents

Data indicates mean ± SD (log10 cells/g of colon contents)

*: *P* < 0.05 versus CN group, **: *P* < 0.01 versus CN group, ***: *P* < 0.05 versus EN group, ****: *P* < 0.01 versus EN group

Collectively, hyperglycemia and endotoxemia didn’t alter the gut microbiota synergically, even though both hyperglycemia and endotoxemia independently modified the proportions of the obligate and facultative anaerobes in the gut microbiota. In some type of bacteria, the effect of hyperglycemia differed between healthy and endotoxemic condition.

## Discussion

The present study demonstrated that persistent hyperglycemia induced by exogenous glucose infusion altered the gut immune functions and microbiota in the colon. Another important finding was that hyperglycemia *per se* could independently alter the mRNA expression levels of inflammatory cytokines in the MLNs. In particular, the mRNA expressions of IL-1β and IL-6 were augmented regardless of endotoxemia condition, and those of CD4^+^ T lymphocyte master genes, such as Gata3 and Foxp3, were increased under healthy condition without endotoxemia in the MLNs, following exposure to prolonged state of hyperglycemic insult for 24 hours. While endotoxemia is known to modulate the distribution of some obligate and facultative anaerobes, including both pathogenic and non-pathogenic bacteria, the present study indicates that hyperglycemia also evoked alterations of the gut microbiota, such as changes in the populations of the *Lactobacillus reuteri* subgroup and staphylococci in the colon under healthy condition.

MLNs, the GALT tissue of greatest consequence, play a key role in the gut and host immune systems [7], and simultaneously regulate both local and systemic inflammatory responses [9]. In the present study, we evaluated the expression of pro-inflammatory cytokines such as TNF-α, IL-1β, and IL-6, representative markers of the early phase of critically ill. The present finding that persistent hyperglycemia for 24 hrs independently up-regulated the mRNA expressions of such inflammatory cytokines except for TNF-α in the MLNs indicates that hyperglycemic state induced by exogenous glucose infusion *per se* can activate the MLNs to modulate the host immune responses. The discharges of TNF-α secretion might have been peaked-out at this moment in this experimental model. While several mechanisms underlying the activation of MLNs by continuous hyperglycemic state could be proposed, macrophages residing in the MLNs [16] are considered as the major source of production of inflammatory cytokines [17]. In a previous study, hyperglycemia over the level of 315 mg/dL could directly impair the functions of activated macrophages, resulting in up-regulation of the mRNA expressions of IL-1β and IL-6 [18]. Collectively, hyperglycemia persisting for a day can augment the inflammatory responses in the MLNs *via* activation of macrophages, thereby modulating the functions of the immune system and organ functions.

CD4^+^ lymphocytes also contribute to the preservation of immune homeostasis and inflammatory responses in the gut [7]. We examined the differentiation of CD4^+^ lymphocytes by measuring the master genes, including the Tbx21 for Th1, the Rorc for Th17, Gata3 for Th2, and Foxp3 for Treg to evaluate whether hyperglycemia and endotoxemia synergistically modulate the balance between pro- and anti-inflammatory responses. Previous study showed that both Th1 and Th17 cells were responsible for the release of inflammatory cytokines [19], whereas Th2 and Treg for the production of anti-inflammatory cytokines such as IL-4 and IL-10 [19, 20]. Contrary to our initial hypothesis, the present study showed that persistent hyperglycemia evoked by exogenous glucose infusion evoked up-regulation of the Th2- (*Gata3)* and Treg (*Foxp3*) master genes in favor of Th1 (*Tbx21*) and Th17 (*Rorc*) in healthy condition, which was not found in the endotoxemic condition. Recent evidence has indicated that T cell function and differentiation, including increased Th1 (inflammatory) to Th2 (anti-inflammatory) differentiation and increased Treg cells were associated with the development of immunosuppression in the late phase of sepsis [21, 22]. Thus, hyperglycemia-induced up-regulation of Th2 and Treg found in our study could, in turn, lead to immunosuppressive status, possibly resulting in the poor outcome in critically ill. Although exact mechanisms to account for hyperglycemia-augmented CD4^+^ lymphocyte differentiation to Th2 and Treg was not clarified in our study, endogenous insulin could affect the differentiation of activated CD4^+^ lymphocytes, leading to the promotion of Th1 to Th2 differentiation [23], and simultaneously enhance the expression of Foxp3, well recognized as a Treg cell marker [24]. Since our model could be hyperinsulinemic due to prolonged state of hyperglycemia, as previously reported [13, 25], the alterations of Gata3 (Th2) and Foxp3 (Treg) expression found in the present study could be indirectly induced through an insulin-dependent pathway.

Disruption of the bacterial community in the gut may be associated with overgrowth of and colonization by pathogenic bacteria, which usually inhabit the intestinal tract in small numbers [26]. While obligate anaerobes belonging to classes such as *Clostridia* and *Bacteroidetes* appear to play pivotal roles in the prevention of dysbiosis, the population of facultative anaerobes, including pathogenic *Enterobacteriaceae,* increases when that of obligate anaerobes is reduced [11]. Indeed, the present study demonstrated that endotoxemia evoked the decrease in the populations of bacteria belonging to the *Bacteroidetes* classe in the colon, while the exact reason for this change remains to be elucidated*.* In lower-level analyses of the gut microbiota, endotoxemia up-regulated the *Clostridium perfringens* while decreasing the *Bacteroides fragilis* subgroups irrespective of hyperglycemic condition. It should be noted that hyperglycemia *per se* evoked alterations of the gut microbiota of the colon even in lower-level analysis, e.g., decrease of the *Lactobacillus reuteri* subgroup and increase of *Staphylococci* in the healthy condition, and decrease of *Enterococcus* and *Enterobacteriaceae* in the endotoxemic condition. While we did not evaluate how these alterations in the colon microbiota were induced, it is plausible to consider that these changes could affect the gut immune function.

Although we demonstrated that inflammatory mediators and differentiation of lymphocytes in MNLs, and microbiological constitute in the intestine were modulated by persistent hyperglycemia and endotoxin in this study independently or dependently, it is mandatory to discuss how these changes interact with each other and consider clinically relevant implications of this study. Hyperglycemia evoked inflammatory cytokines while promoting differentiation to Th2 and Treg which are responsible for the release of anti-inflammatory cytokines in MNLs. Although these changes seem to be contradictory, it is often the case that hyper-inflammatory and anti-inflammatory responses coexist in the disease condition such as sepsis in which hyper-inflammatory condition is followed by anti-inflammatory response at the almost same time [27]. Thus, it is likely that the alteration of immune status induced by hyperglycemia contributed to the deterioration of the gut immune function, leading to the alteration of the gut microbiota. On the other hand, endotoxemia evoked the alteration of the gut microbiota without the effects on inflammatory cytokines and CD4^+^ T cells differentiation in MNLs. Although the true mechanism was not elucidated in this study, some possibilities should be considered. One mg/kg LPS, which was less than the dose of 4 mg/kg we used in the previous study [14], might not be enough to evoke the alteration of cytokine production and CD4^+^ T cells differentiation, even though the survival rate of the EH group at 24 hours was about 80 % in our preliminary study, which implies the severity of disease condition induced by this dose of LPS. Otherwise, concentration of cytokine had already peaked out and the alteration of CD4^+^ T cells differentiation had already finished at 24 hours. Further study is warranted to elucidate the mechanism how these changes interact and modulate the gut immune function. While the true mechanism was not elucidated in this study, the alteration of the gut microbiota, which was induced by hyperglycemia and endotoxemia in this study, could evoke the deteriorated gut immune function and bacterial translocation into systemic circulation [28]. Considering the results of this study, an intervention should be taken into account to maintain the homeostasis of the microbiota and prevent the gut immune dysfunction in patients with uncontrolled hyperglycemia and endotoxemia, such as administration of probiotics. Previous study demonstrated that the *Lactobacillus reuteri* subgroup considered as one of probiotic bacterial strains, which was reduced by hyperglycemia in this study, may be able to depress growth, adherence and virulence of *Staphylococci* [29, 30]. It is expected that new interventions are developed to maintain the intestinal microbiota in critically ill patients in future studies.

Some limitations of this study must be borne in mind while interpreting the data herein. *First*, some may argue that we should have measured the respective protein concentrations directly caused by the alterations of cytokine mRNA. Although previous study showed that protein level was remarkably elevated as well as cytokine mRNA in an endotoxemic model [31], the protein concentrations are not necessarily dependent on the changes of cytokine mRNA. Another issue is the semi-quantitative strategy applied may provide only a limited view of the ecological disruption potentially caused by endotoxemia. Since the gut microbiota varies widely in composition among individuals even in health depending on their geographical origin and/or location within the gut, further examination of intestinal environments, such as of organic acids, may be required to determine the abnormal changes of the microbiota. *Second*, persistent hyperglycemia observed in critically ill patients is basically not caused by continuous glucose administration but by endogenous alterations such as resistance to insulin [2]. However, we used exogeneous glucose infusion model to mimic clinical situations since it is difficult to maintain stable hyperglycemia level in rodent models without exogeneous glucose, and exogeneous glucose infusion, such as parenteral nutrition, was often performed in the intensive care unit, while LPS model did not introduce hyperglycemia in this study. Furthermore, there are a large number of confounding variables in clinical settings, such as antibiotic use, starvation, enteral feeding and others which trigger alterations of the gut microbiota. Ultimately, the effects of oral antibiotics or probiotics on gene expression in the MLNs should be settled by further study. After all, in addition to having different microbiota from humans, it may not be possible to precisely mimic clinical situations in rodent models under experimental conditions. *Third,* the values of CD4^+^T cells, evaluated by the change of transcriptional factors through RT-PCR, could not reflect the actual number of each lymphocyte since extraction of m-RNA was performed from the contents of MNLs without cell sorting by FACS, and the alteration of transcriptional factors could not always be consistent with the number of lymphocytes. Although these values of CD4^+^T cells could not represent the actual number, the trend of CD4^+^ T cells differentiations could be detected by this measurement method. Finally, we performed lots of multiple comparisons to evaluate the alteration of bacteria in colon microbiota, which might lead to the false positive. However, the possibility of false positive could be a minimum, since we performed multiple comparisons after detection of interaction between two factors, hyperglycemia and endotoxemia.

## Conclusions

In conclusion, the present study showed that persistent hyperglycemia evoked by exogenous glucose infusion for 24 hours modulates both inflammatory responses and CD4^+^ T cells differentiation in the MLNs, and induces the alterations of the colon microbiota. Further study is warranted to elucidate the causal relationship between persistent hyperglycemia and altered gut immunity as well as clinical implications of these changes in gut.

## Additional file

Additional file 1: Figure S1 The classification of bacteria in the colon microbiota. This figure represents the classification of colon bacteria which was evaluated in this study. (TIFF 86 kb)
