# Supplementary figures and images for: Persistent hyperglycemia modulates gut immune function and microbiota in rats
Source: J Intensive Care. 2015 Jul 23;3(1):34. doi: 10.1186/s40560-015-0101-8 (PMC4511975; doi:10.1186/s40560-015-0101-8)

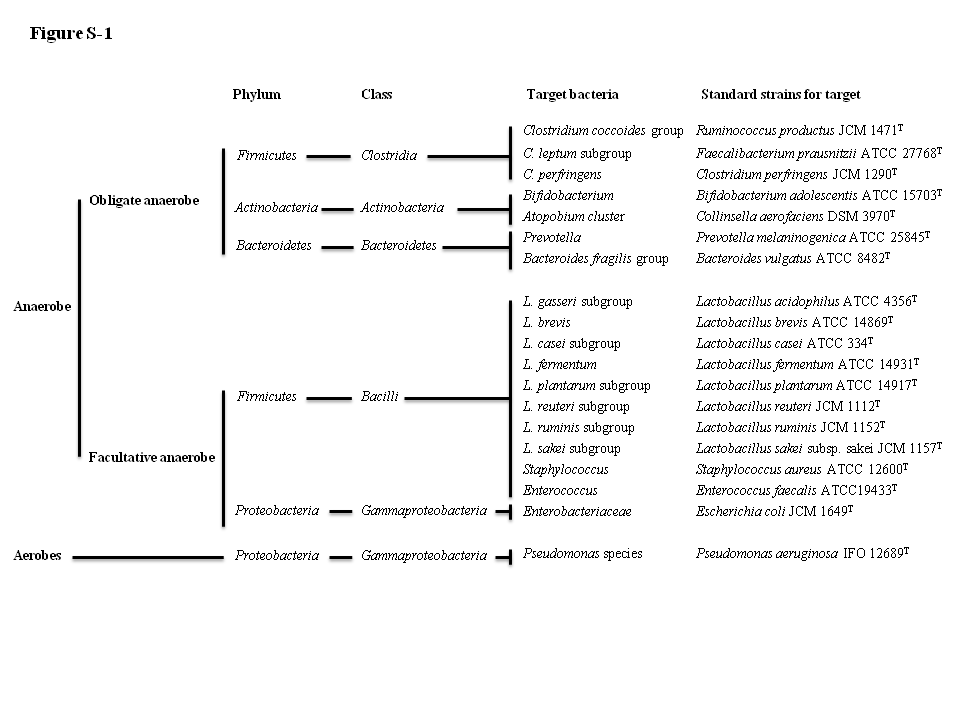

Supplement: Additional file 1: Figure S1 — The classification of bacteria in the colon microbiota. This figure represents the classification of colon bacteria which was evaluated in this study. (TIFF 86 kb) [file 40560_2015_101_MOESM1_ESM.tiff]
